# Monitoring melanoma recurrence with circulating tumor DNA: a proof of concept from three case studies

**DOI:** 10.18632/oncotarget.26451

**Published:** 2019-01-04

**Authors:** Ashleigh C. McEvoy, Michelle R. Pereira, Anna Reid, Robert Pearce, Lester Cowell, Zeyad Al-Ogaili, Muhammad A. Khattak, Michael Millward, Tarek M. Meniawy, Elin S. Gray, Melanie Ziman

**Affiliations:** ^1^ School of Medical and Health Sciences, Edith Cowan University, Joondalup, Western Australia 6027, Australia; ^2^ Level1 Melanoma Clinic, Hamilton Hill, Western Australia 6163, Australia; ^3^ Department of Molecular Imaging and Therapy Service, Fiona Stanley Hospital, Murdoch, Western Australia 6150, Australia; ^4^ Department of Medical Oncology, Fiona Stanley Hospital, Murdoch, Western Australia 6150, Australia; ^5^ School of Medicine and Pharmacology, The University of Western Australia, Crawley, Western Australia 6009, Australia; ^6^ Department of Medical Oncology, Sir Charles Gairdner Hospital, Nedlands, Western Australia 6009, Australia; ^7^ Centre for Ophthalmology and Visual Science, University of Western Australia, Crawley, Western Australia 6009, Australia; ^8^ School of Biomedical Sciences, University of Western Australia, Crawley, Western Australia 6009, Australia

**Keywords:** circulating tumor DNA, ctDNA, melanoma, recurrence, droplet digital PCR

## Abstract

**Background:**

A significant number of melanoma patients experience recurrence to distant sites, despite having had surgical treatment of the primary lesion, with curative intent. Monitoring of patients for early evidence of disease recurrence would significantly improve management of the disease, allowing timely therapeutic intervention. Circulating tumor DNA (ctDNA) is becoming a well-recognized biomarker for monitoring malignancies and has, in a few studies, been shown to signify disease recurrence earlier than conventional methods.

**Methods:**

We performed a retrospective analysis of plasma ctDNA using droplet digital PCR (ddPCR) in 30 primary melanoma patients with tumors harboring BRAF, NRAS or TERT promoter mutations. Mutant specific ctDNA, measured during clinical disease course, was compared with disease status in patients with confirmed disease recurrence (*n* = 3) and in those with no evidence of disease recurrence (*n* = 27).

**Results:**

Mutant specific ctDNA was detected in all three patients with disease recurrence at the time of clinically confirmed progression. In one case, plasma ctDNA detection preceded clinical identification of recurrence by an interval of 4 months. CtDNA was not detected in patients who were asymptomatic and had no radiological evidence of recurrence.

**Conclusions:**

This study demonstrates promising results for the use of ctDNA as an informative monitoring tool for melanoma patients having undergone tumor resection of an early stage primary tumor. The clinical utility of ctDNA for monitoring disease recurrence warrants investigation in prospective studies as it may improve patient outcome.

## INTRODUCTION

Whilst the majority of melanoma patients are cured by surgical resection, approximately 30% of patients will present with systemic recurrence at some point during their lifetime [1], sometimes 10 or more years after diagnosis of primary melanoma [2–7]. Numerous factors such as clinical staging as well as host and tumor-dependent variables have been associated with recurrence [1, 7, 8]. Such characteristics however are not infallible and do not account for all patients that present with recurrence [4]. The risk of melanoma recurrence therefore is never entirely eliminated and the ability to correctly identify a subset of patients at risk of recurrence from a patient population with an expected favorable outcome is therefore challenging.

Timely detection of recurrence is of significant importance for improved patient outcomes given that surgical removal of solitary or a small number of isolated metastases most often improves overall survival [9–11]. Moreover, it has recently been shown that metastatic melanoma patients are more likely to achieve a long lasting response to systemic therapy when it is administered to patients with a low burden of disease [12, 13]. Currently, sentinel lymph node biopsies (SLNB), physical examination and radiological imaging methods such as ^18^F-FDG PET/CT are employed to stage primary melanomas and detect metastatic disease. Whilst ^18^F-FDG PET/CT provides valuable information on the location and metabolic activity of metastatic disease through real-time whole-body imaging [14], such imaging is not routinely used in the follow-up of early stage patients (American Joint Committee on Cancer (AJCC) stages 0-IIA) but rather for staging symptomatic patients or monitoring high-risk patients [15]. SLNBs are occasionally recommended for staging purposes for those with ≥1 mm thick melanomas [16]. However, they are relatively invasive and prohibitively costly for ongoing routine monitoring [17, 18]. Consequently, physical examination (including lymph node palpation) and radiological assessments are the primary surveillance strategies for patients having undergone primary tumor resection [15]. There is however, little consensus on the frequency of clinical assessment; the National Comprehensive Cancer Network (NCCN) guidelines suggest physical examinations be conducted every 3 to 12 months for the first 5 years and then annually thereafter for AJCC stage IA-IIA, and every 3 to 6 months for 2 years, then every 3 to 12 months for 3 years followed by annual checks and optional imaging for stages IIB to IV [15]. Importantly, physical examinations are usually inefficient at detecting distant metastases (disease that has spread to other sites such as lung, liver, bone or brain) [17], and thus melanoma recurrence is often detected at a late stage. Thus, there remains a need to improve follow-up for early detection of melanoma recurrence, which could lead to timely interventions and earlier treatment options that may improve patient outcomes.

CtDNA is an easily accessible marker that may be suitable for routine monitoring of disease recurrence [18–22]. The exact mechanism of ctDNA production remains elusive but it is thought to be released from cells predominantly by apoptosis and possibly through active secretion [23, 24]. Recent studies show that ctDNA levels exhibit a close correlation with disease status in a number of different cancers including gastric [25], colorectal [26], colon [27] and breast [19, 28], although, most studies have been conducted in the metastatic setting where ctDNA is likely to be relatively abundant. A few studies have demonstrated a potential utility for detecting ctDNA post-surgery in early stage cancers [29]. The presence of ctDNA was found to correlate with minimal residual disease in breast [28] and colon [27] cancers, and provide an early measure of clinically detectable recurrence in early stage lung cancer [30].

While there is now growing evidence that ctDNA has the potential to detect disease recurrence earlier or at the same time as conventional methods [27, 28, 30–32], the use of ctDNA for monitoring of residual disease and recurrence in clinically disease-free patients remains limited [27, 30, 31]. Thus, it remains unclear whether ctDNA is detectable at early stages and/or can provide early evidence of disease progression.

In this study, we aimed to determine whether in melanoma, ctDNA could indicate the presence of disease recurrence and/or of metastatic disease, earlier than conventional methods. To do this, we analyzed *BRAF*, *NRAS* and *TERT* promoter mutant ctDNA using serial blood samples from melanoma patients with and without confirmed disease recurrence.

## RESULTS

### Patient samples and cohort summary

**Figure 1:**
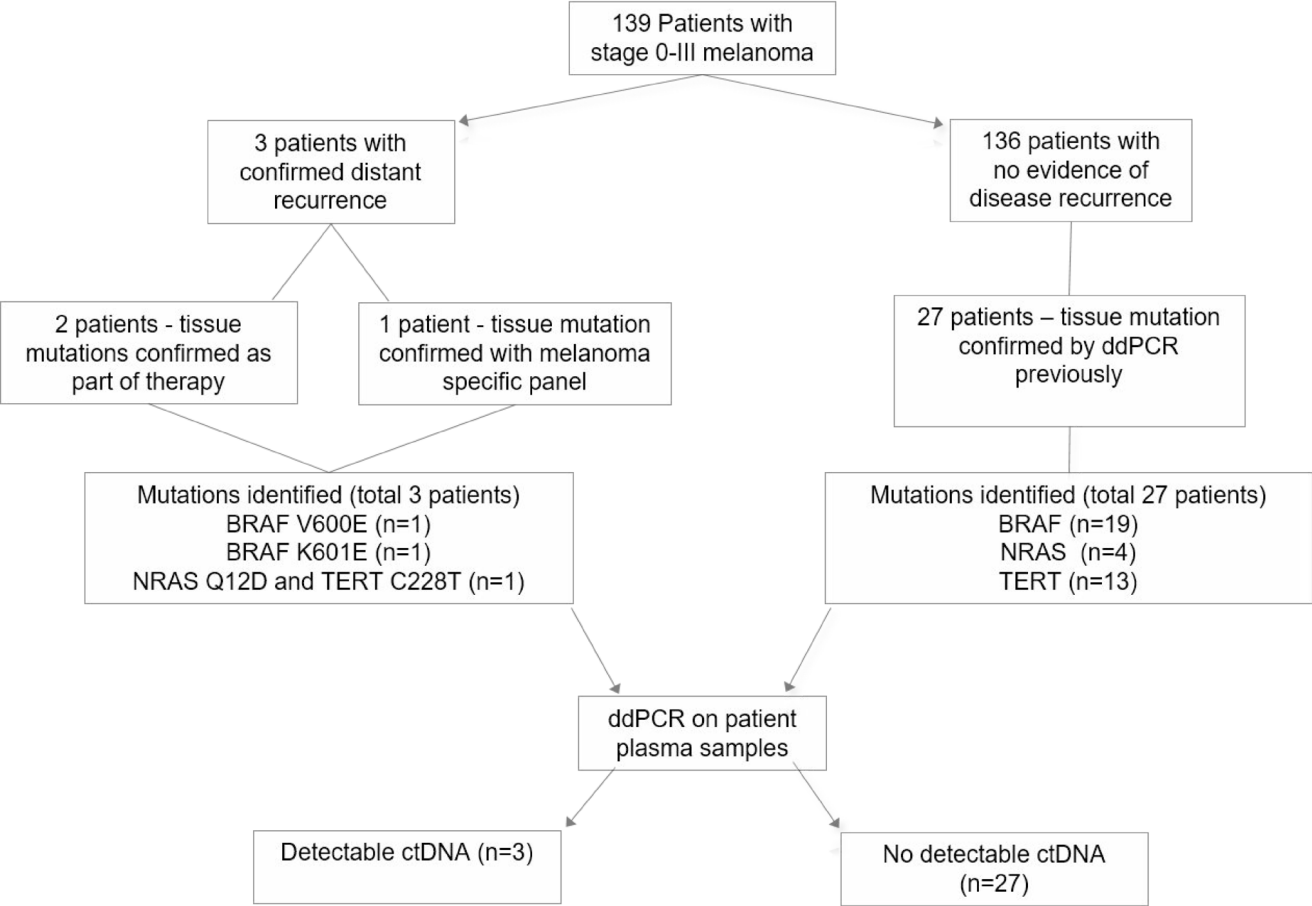
Overview of patient enrolment, sample collection and analysis CtDNA was detectable in all three patients with disease recurrence but not in patients with no evidence of disease recurrence.

**Figure 2:**
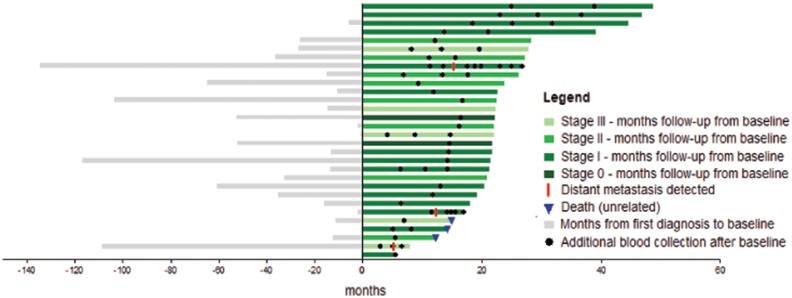
Swimmers plot in months from diagnosis of first primary melanoma to last follow-up A total of 30 patients were assessed in the study. Bar colors indicate the melanoma stage at enrolment. All patients had a blood sample drawn at enrolment (zero months) and at the time points indicated (black dots). Three patients experienced recurrence during study follow up (red line) and another three cases died from reasons unrelated to melanoma (blue triangle).

From the original cohort of 139 patients, we selected 3 patients who developed metastatic disease and 27 patients with no evidence of disease recurrence at last follow-up or at the time of unrelated death for retrospective analysis (Figures 1 and 2). The median age was 63.7 (range 28 to 92 years). The median time from primary melanoma resection to enrolment was 14.7 months (range 0 to 134.6 months) and the median follow-up was 22 months (range 5.9 to 48.8 months).

Nineteen patients harbored a *BRAF* mutation, V600E (*n* = 8), V600K (*n* = 10) or K601E (*n* = 1); 4 patients had *NRAS* mutations, Q61K (*n* = 3), Q12D (*n* = 1); 13 had *TERT* promoter mutations, C228T (*n* = 8), C250T (*n* = 5) and 10 patients had more than one mutation. Of the patients that presented with disease recurrence, patient 2 harbored the *BRAF* K601E mutation, patient 6 harbored *NRAS* Q12D and *TERT* C228T mutations and patient 23 harbored the *BRAF* V600E mutation. Patient characteristics and their mutation status are described in Table 1.

**Table 1:** Patient and primary tumor characteristics

Patient number	Sex	Age at diagnosis	AJCC stage	Classification	Breslow (mm)	Ulceration	Mitoses (rate per sq.mm)	Regression	Mutation^*^
1	F	38	I	Unclassified	0.35	-	-	Yes	BRAF V600E
**2**	**M**	**50**	**III**	**Nodular**	**8.3**	**Incipient**	**4**	**No**	**BRAF K601E**
3	M	90	II	SSM	3.7	Yes	10	No	TERT C228T
4	M	83	I	SSM	1	No	1	Slight	BRAF V600E, NRAS Q61K, TERT C228T
5	M	93	III	-	-	-	-	-	TERT C228T
**6**	**M**	**81**	**I**	**Unclassified**	**1.1**	**No**	**No**	**No**	**NRAS Q12D, TERT C228T**
7	M	75	I	CBD	1.2	No	No	Yes	NRAS Q61K, TERT C250T
8	F	28	I	SSM	0.38	No	<1	No	BRAF V600E
9	M	46	I	SSM	0.45	No	No	Yes	BRAF V600K
10	M	89	II	Nodular	5.5	No	6	-	BRAF V600K, TERT C250T
11	F	92	I	SSM	0.3	No	No	No	BRAF V600K
12	F	45	I	Unclassified	0.3	-	-	No	BRAF V600K
13	M	38	I	SSM	0.3	No	No	No	BRAF V600E
14	M	41	0	-	-	-	-	-	TERT C228T
15	M	73	III	-	-	-	-	-	BRAF V600K, TERT C250T
16	M	58	II	SSM	7	No	No	No	NRAS Q61K
17	M	56	0	-	-	-	-	-	BRAF V600E
18	M	58	III	-	-	-	-	-	NRAS Q61K, TERT C228T
29	M	44	II	SSM	1	Yes	Active	No	TERT C250T
20	F	43	I	Unclassified	0.5	No	No	No	BRAF V600K
21	M	67	II	Nodular	25	Yes	5	No	BRAF V600E, TERT C228T
22	M	74	II	Nodular	2	Yes	10	Yes	BRAF V600E, TERT C250T
**23**	**M**	**62**	**I**	**Nodular**	**1.82**	**-**	**20**	**-**	**BRAF V600E**
24	M	75	II	Lentigo	2.1	No	8	Yes	BRAF V600K
25	M	90	III	-	-	-	-	-	BRAF V600K
26	M	88	II	SSM	0.4	-	-	Yes	TERT C228T
27	M	69	I	SSM	0.4	No	No	No	BRAF V600K
28	M	58	I	SSM	0.45	No	No	Yes	BRAF V600K
29	F	68	I	Unclassified	0.3	-	-	Yes	BRAF V600E
30	M	40	I	Unclassified	0.45	-	-	Yes	BRAF V600E

Abbreviations: SSM = Superficial spreading melanoma; CBD = cannot be determined; – = unavailable. ^*^Assessed by ddPCR

Patients 2, 6 and 23 presented with disease recurrence and had their tumors assessed for mutations by NGS sequencing in addition to ddPCR analysis

Patients indicated in bold are those that suffered recurrence.

### Quantification of mutant specific ctDNA from plasma

**Figure 3:**
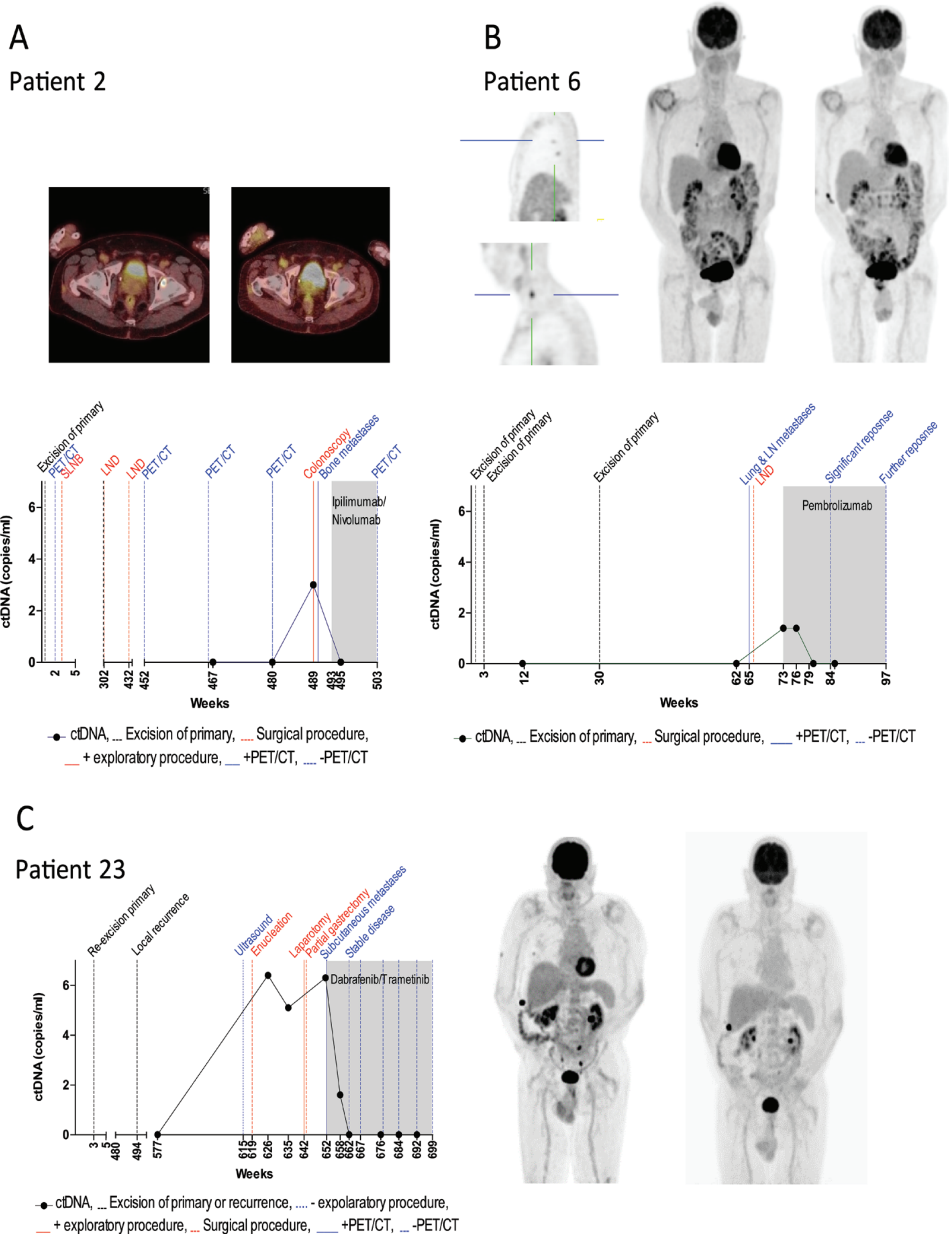
Monitoring ctDNA levels during clinical disease course The levels of plasma ctDNA for three melanoma patients presenting with disease recurrence is represented in relation to the patients’ clinical history in weeks from first primary diagnosis. CtDNA concentrations were defined by (**A**) *BRAF* K601E (patient 2), (**B**) *NRAS* Q12D (patient 6) and (**C**) *BRAF* V600E (patient 23) mutant copies. The dotted black line indicates the time of resection of primary melanoma or local recurrence. Positive and negative radiological outcomes are represented by a solid and dashed blue line, respectively. Surgical procedures are represented by dashed red lines. Solid and dotted red lines represent positive and negative exploratory procedures respectively. The shaded area indicates the period during which systemic therapy was administered.

Overall, cfDNA was isolated from 73 plasma samples taken from 30 patients. The number of mutant ctDNA copies per mL of plasma was quantified by ddPCR. CtDNA was measurable in all 3 patients (Figure 3) that presented with disease recurrence and in none of those without recurrence at last follow-up.

### Patient 2

Curative resection was performed for a primary nodular melanoma located on the neck with a Breslow measurement of 8.35mm. PET/CT and SLNB conducted immediately after resection revealed no regional or distant metastases. The patient was disease free for approximately five years, then presented with two positive lymph node (LN) metastases at 302 weeks and with one positive LN at 432 weeks after primary diagnosis. All positive LNs were resected at time of identification. Approximately 36 weeks after the last resection the patient was recruited into this study at which time ctDNA was not detected (Figure 3A). 13 weeks later (48 weeks post lymph node dissection (LND)), PET/CT revealed no evidence of metastases and ctDNA remained negative. Following an investigative colonoscopy one-year post LND and 9 weeks post negative PET/CT, a subsequent biopsy revealed stage IV melanoma with bowel metastasis at which time ctDNA was detected at 3 copies/mL of plasma. A subsequent PET/CT also revealed bone metastases. The patient commenced full dose combination Ipilimumab/Nivolumab therapy and within three weeks, ctDNA was undetectable. At last follow-up (week 503), the patient had no evidence of metastatic disease on PET/CT.

### Patient 6

Patient 6 underwent curative resection for a 1.1 mm primary melanoma on the right anterior scalp, with a mitotic rate of 2/sq. mm but no ulceration, regression, perineural or lymphovascular invasion. Re-excision was performed at week 3. At week 12 the patient was recruited into the study and ctDNA was not detected (Figure 3B). Approximately 30 weeks following initial diagnosis, a second primary invasive melanoma on the scalp measuring 3.1 mm with vascular involvement was excised with a wide margin. At week 62, ctDNA was not detected, but clinical and radiological evaluation three weeks later revealed a level III cervical lymph node (which was confirmed as metastatic melanoma on pathology) and five FDG avid nodules in the right lung (largest measuring 6 mm). No blood was collected at this time. Prior to commencing treatment of pembrolizumab almost eight weeks later, *NRAS* Q12D mutant ctDNA was detected at 1.4 copies/mL of blood. Within 3 weeks of commencing treatment, ctDNA was undetectable. Following PET/CT at week 84 post initial diagnosis, a significant but incomplete improvement was observed when compared to the previous PET/CT scan and ctDNA was not detected. The patient subsequently showed complete response on PET/CT and eighteen months post treatment cessation, remains disease free. The *TERT* promoter mutation found in the tumor, was not detected in plasma at any time point in this patient.

### Patient 23

In 2004, the patient underwent resective surgery for a primary nodular melanoma on the left thigh. Histology reported the melanoma contained an unusual polypoidal appearance however demonstrated invasion of the reticular dermis (Clark level 4) and a maximum thickness of 1.82 mm. The lesion extended to within 1mm of a lateral resection margin and hence further wide local excision was recommended. Re-excision was completed within 3 weeks with no further residual tumor identified. There was at least 10 mm of skin and subcutaneous fat at either edge of the scar. Nodules of metastatic melanoma were identified and excised from the dermis and subcutis within and adjacent to the scar site 494 weeks after the primary melanoma was identified. Excision was complete with clearance of 0.5 mm from the deep and side margins. At week 577 the patient was recruited into the study and ctDNA was not detected (Figure 3C). Abdominal ultrasound at week 615 showed no evidence of metastatic disease. Approximately 42 weeks later at week 619, the patient presented with a red, painful eye and ocular melanoma was found. The patient elected to undergo enucleation. We did not have a blood sample to test ctDNA at this time point. Histological and molecular analysis of the tumor indicated the presence of a *BRAF* V600E mutation. *BRAF* V600E mutant ctDNA was detected at 6.4 copies/mL of plasma at week 626, and again at week 635 at 5.1 copies/mL of plasma. Having refused PET/CT scans due to claustrophobia, the patient underwent a gastroscopy for anemia at week 642, which revealed a 4 cm gastric metastasis and 2 ileum metastases (10 cm apart). All metastases were surgically removed following confirmation of metastatic melanoma on biopsy. Histological and molecular analysis of the gastric tumor also indicated the presence of a *BRAF* V600E mutation. No blood samples were collected at this time. At week 652 PET/CT revealed progressive disease with bone metastases. At this time, ctDNA was detected at 6.3 copies per mL of plasma and combination therapy of dabrafenib and trametinib was commenced. CtDNA levels declined to 1.6 copies/ml at 6 weeks and were undetectable at 10 weeks post treatment commencement. At week 667 (14 weeks post treatment initiation), PET/CT revealed complete response which has persisted and ctDNA levels have remained undetectable.

## DISCUSSION

This report describes the results of a retrospective assessment of mutant specific ctDNA for patients with cutaneous melanoma after removal of a primary lesion (*n* = 30). In three patients with disease recurrence, ctDNA was detectable at the time of clinically confirmed disease recurrence. In one case, plasma ctDNA detection preceded clinical identification of recurrence by an interval of 4 months. By contrast, ctDNA was not detected at any time point in patients with no clinical evidence of disease recurrence.

Although there was variation in the timing of plasma collections, which did not necessarily coincide with functional imaging, three plasma samples were available for each recurring patient before the initiation of systemic therapy. In this study, no mutant specific ctDNA was detected in any patient prior to detection of metastatic disease, however ctDNA was later detected in all three patients that presented with disease recurrence and subsequently diagnosed with stage IV metastatic melanoma. This is in contrast with previous studies in stage II and III melanoma [33], uveal melanoma (Beasley), breast [28] and colon [27] cancers, which showed that ctDNA was detectable in patients with early stage disease.

In patient 23, *BRAF* V600E mutant ctDNA was detectable 4 months prior to metastatic disease being clinically evident. Notably, this patient was not undergoing routine PET/CT scans due to severe claustrophobia, and metastatic disease was discovered during a gastroscopy performed to evaluate asymptomatic anemia. Importantly however, this suggests that ctDNA may serve as an alternative option to those not wishing to undertake routine PET/CT scans.

Although we detected *NRAS* Q12D mutant ctDNA at 1.4 copies per mL of plasma at one time point in patient 6, we did not detect *TERT* C228T mutant ctDNA at any time point in this patient, although it was detected in the tumor. This may be explained by the fact that *TERT* promoter mutations demonstrate lower copy numbers relative to other driver mutations [34] (Calapre *et al*., manuscript submitted). Moreover, *NRAS* mutant ctDNA was not detected in the plasma sample collected three weeks prior to PET/CT which identified pulmonary metastases, nor was it found in the blood sample collected after initiation of systemic therapy where the PET/CT scan revealed a significant but incomplete response. This highlights a limitation in the sensitivity of our ctDNA detection approach which may be improved with increasing the amount of plasma from which cfDNA is extracted, as well as by introducing pre-amplification of cfDNA prior to ctDNA quantification. The use of next generation sequencing custom panels using unique molecular identifiers may also improve sensitivity as well as enable monitoring of a large number of mutations. Previously, we have shown a correlation between ctDNA and quantitative metabolic tumour burden (MTB) derived from PET/CT in treatment naïve patients [35]. In addition, future studies may further benefit from a correlation between ctDNA and MTB in patients undergoing treatment as this will further indicate the limit at which ctDNA can be detected in patients presenting with distant metastasis(es).

Of importance is the evidence of distant metastatic disease in patient 2 almost 9 years after the primary melanoma was resected and more than 3 years after stage III disease was initially diagnosed which supports the evidence that recurrences have been observed many years after primary resection [2, 3, 6]. During this time, 2 PET/CT scans were performed, and it was ultimately the colonoscopy which detected metastatic disease and prompted the third PET/CT scan which highlights the need for improvements in routine monitoring.

Whilst stage III melanoma patients have a higher risk of recurrence than stage I [36, 37], in our cohort two of the patients with stage 1 disease and only 1 of the patients with stage III disease at time of diagnosis of the primary, developed metastatic disease. Furthermore tumor thickness has been considered to be an important prognostic risk factor for recurrence [38], however here we report recurrence from primary tumors of 1.1 mm, 1.83 mm and 8.3 mm thickness. Previous studies that have compared time to recurrence with clinical staging as well as host and tumor-dependent variables, have shown that older age, gender (male), thin and non-ulcerated melanomas are each independently associated with a late recurrence (>8 years from time of diagnosis) [7, 8]. In our study, we report distant recurrence in one patient with a 1.1 mm thick non-ulcerated primary 63 weeks after initial diagnosis (Figure 3B). Conversely, Faries *et al*. reported tumors less than 1.23 mm and the lack of ulceration to each be independently associated with a late recurrence which they defined as >10 years from initial diagnosis [7]. In one patient with a 1.83mm thick primary, we report a local recurrence more than 9 years and metastatic disease more than 12 years after initial diagnosis (Figure 3C), however Faries *et al.* reported thicker tumors (2.93 mm) to be independently associated with an early recurrence (within 3 years of diagnosis) [7]. Whilst we only report 3 recurrences, the heterogeneity between patient outcomes and tumor-dependent variables highlight the challenges associated with correctly identifying a subset of patients at risk of recurrence from a patient population with an expected favorable outcome.

The major limitations of this study are the relatively short follow up time and the low number of patients analyzed, hence, the low number of observed recurrences. Nevertheless, we provide a proof of concept indicating that monitoring ctDNA during follow-up of melanoma patients after surgery may provide a potentially useful clinical test. Our experience confirms that ctDNA surveillance provides a supplementary method of monitoring patients without the additional risk of radiation exposure, invasiveness, and high cost of radiological scan, and that ctDNA tests indicate disease recurrence at least at the time of clinical confirmation. The detection of disease recurrence earlier may provide an increased window of opportunity for intervention that may positively impact on the patient's outcomes. Furthermore, ctDNA testing may offer an alternative option for routine monitoring for patients who are unable, for whatever reason, to undergo functional imaging. Future prospective studies are needed to demonstrate the utility of ctDNA for routine monitoring of early stage melanoma patients.

In conclusion, this study has provided proof-of-concept evidence for the prospective and frequent monitoring of ctDNA in melanoma patients. However, prospective studies and improved assay sensitivity are needed before it can be used for monitoring patients after resection of their primary for early detection of metastatic disease.

## MATERIALS AND METHODS

### Patient and sample collection

Patient enrolment and study overview are presented in Figure 1. We analyzed a group of patients that were prospectively accrued into the “Blood Based Biomarkers: Prognostic Tools for Melanoma Recurrence” project between July 2013 and February 2017. The study was approved by the Human Research Ethics Committee of Edith Cowan University under reference numbers 11543 and 13313 and Sir Charles Gairdner Hospital (no.2013-246). A cohort of 139 melanoma patients (stage 0–III), diagnosed and resected in the previous 10 years were eligible for enrolment. A blood sample was collected at enrolment and then again between 3–25 months thereafter. Overall, three cases experienced distant disease recurrence during the sample collection period. The *BRAF* mutation status of these three tumors was determined previously [38] and by Next Generation Sequencing (NGS) using a custom targeted sequencing panel for a third case which was found to be *BRAF* wild-type. A subset of patients (*n* = 27) with no clinical evidence of disease recurrence at last follow-up was selected for ctDNA assessment. The main criteria for their selection was based on the availability of primary tumor mutational data determined as part of a previous study [39] in which they were found to be positive for at least one of the common melanoma mutations (*BRAF*, *NRAS*, *TERT*).

### Isolation and quantification of circulating tumor DNA from plasma

Blood samples were collected into EDTA vacutainer tubes and stored at 4° C. Plasma was separated within 24 hours by centrifugation at 1600 g for 10 minutes, followed by a second centrifugation at 2000 g for 10 minutes, then stored at −80° C until extraction. Cell free DNA (cfDNA) was isolated from 5 mL of plasma using the QIAamp Circulating Nucleic Acid Kit (Qiagen) as per the manufacturer's instructions, eluted in 40 μl AVE buffer (Qiagen) and stored at –80° C until ctDNA quantification. CtDNA was quantified by ddPCR targeting the specific mutation found in the patient's tumor. *BRAF* mutations were detected using in-house primer/probe sets [40], *NRAS* mutations were tested using PrimePCR assays (Bio-Rad) and *TERT* promoter mutations were detected using in-house primer/probe sets [41]. Amplifications were carried out in a 20 μL reaction mixture containing ddPCR Supermix (Bio-Rad), 900 nM primers, 250 nM of each probe and 5 ul (for *TERT* assay) or 8 μL (for *BRAF/NRAS* assays) cfDNA. Droplets were generated using the Automatic Droplet generator QX200 AutoDG (Bio-Rad). Droplets were analyzed through a QX200 droplet reader (Bio-Rad) and data was analyzed using QuantaSoft analysis software (Bio-Rad). For quantification, a minimum of 10,000 acceptable droplets was required per 20 uL reaction. To ensure the accuracy of results, each sample was tested minimally in duplicate with each run including a non-template control and gDNA from cell lines containing the relevant mutation. Samples were deemed positive according to criteria previously described [40, 41].

In addition, given that 82% of benign melanocytic nevi carry *BRAF* mutations [42], we tested a cohort of healthy controls with ≥50 nevi for *BRAF* V600E and *BRAF* V600K mutant specific cfDNA (Supplementary Table 1). None of the samples were found positive, suggesting that *BRAF* V600E/K cfDNA is specific to tumor derived DNA and/or nevi do not release sufficient mutated DNA to be detectable in plasma using these assays.

## SUPPLEMENTARY MATERIALS FIGURES AND TABLES


