# Supplementary material for: Monitoring melanoma recurrence with circulating tumor DNA: a proof of concept from three case studies
Source: Oncotarget. 2019 Jan 4;10(2):113–22. doi: 10.18632/oncotarget.26451 (PMC6349444; doi:10.18632/oncotarget.26451)
Supplement: Supplementary file 1 [file oncotarget-10-113-s001.pdf]

Monitoring melanoma recurrence with circulating tumor DNA: a proof of concept from three case studies

SUPPLEMENTARY MATERIALS

Supplementary Table 1: Plasma mutant DNA analysis in individuals with high nevi counts

ID	BRAF V600E Copies/mL	BRAF V600K Copies/mL
CAM 003	0	0
AM 008	0	0
CAM 031	0	0
CAM 034	0	0
CAM 035	0	0
CAM 047	0	0
CAM 049	0	0
CAM 050	0	0
CAM 051	0	0
CAM 058	0	0
AM170	0	0
AM172	0	0
AM191	0	0
